# Supplementary material for: Evaluation of SARS-CoV-2 ORF7a Deletions from COVID-19-Positive Individuals and Its Impact on Virus Spread in Cell Culture
Source: Viruses. 2023 Mar 21;15(3):801. doi: 10.3390/v15030801 (PMC10051148; doi:10.3390/v15030801)
Supplement: Supplementary file 1 [file viruses-15-00801-s001.zip › viruses-2192095-supplementary.pdf]

Figure S1 Short Tandem Repeats (STRs) contributors analysis of 16538_ORF7aΔ365 sample

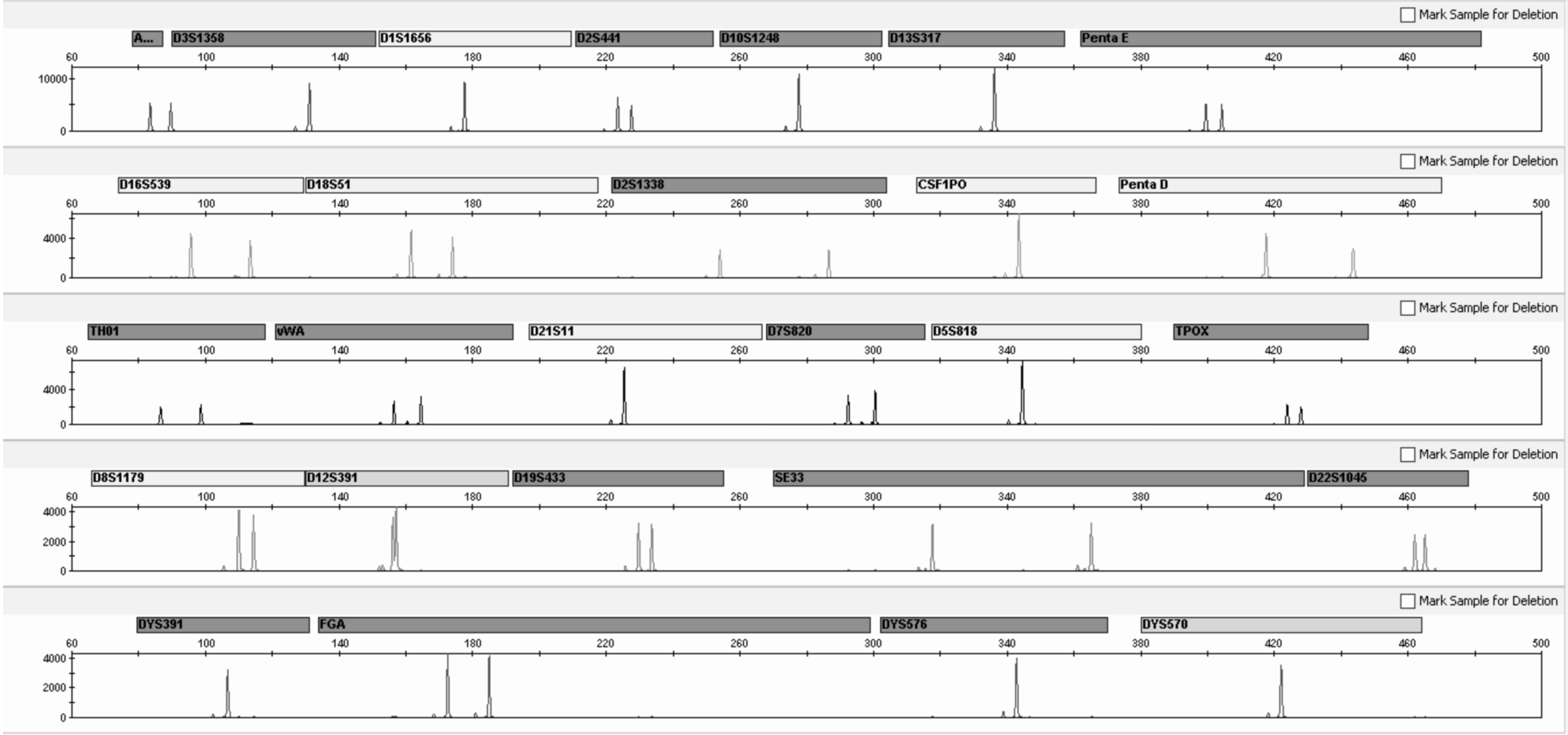

Figure S2: Visualization of sgRNAs on 2% agarose gels. **A**) Position of the primers used for cDNA -PCR amplification, forward primer (Leader -18-41) and reverse primers located in ORF7a (27511-27491 and 27531-27512); ORF8 (28003-27986). **B**)Primer pairs, 1, 2 and 3, used for PCR reaction and the corresponding amplification products run in agarose gels staining with ethidium bromide. Clinical samples template are indicated in the top of the gel. Sample 16538_ORF7a_Δ365 displays the same profile as the wt-sample (16305_ORF7a_wt) due to the presence of the counterpart wt version of the virus. **C**) Amplified sgRNAs from clinical samples using primer pair SARS-Leader 18-41F + N_28529-28508R and visualized through microfluidic electrophoresis. Clinical samples: 1-11785_ORF7a_wt, 2-58301_ORF7a_Δ190, 3-16553_ORF7a_wt, 4-16991_ORF7aΔ339, 5- 16538_ORF7aΔ365 and the counterpart_wt viruses**.** The bands indicated by red arrow were excised from agarose gel (E-gel 2%) and Sanger sequenced. Canonical sgRNA are shown in Fig 3. L ladder, Blue arrows: ORF8; Yellow arrows: ORF7a; Orange arrows: N.

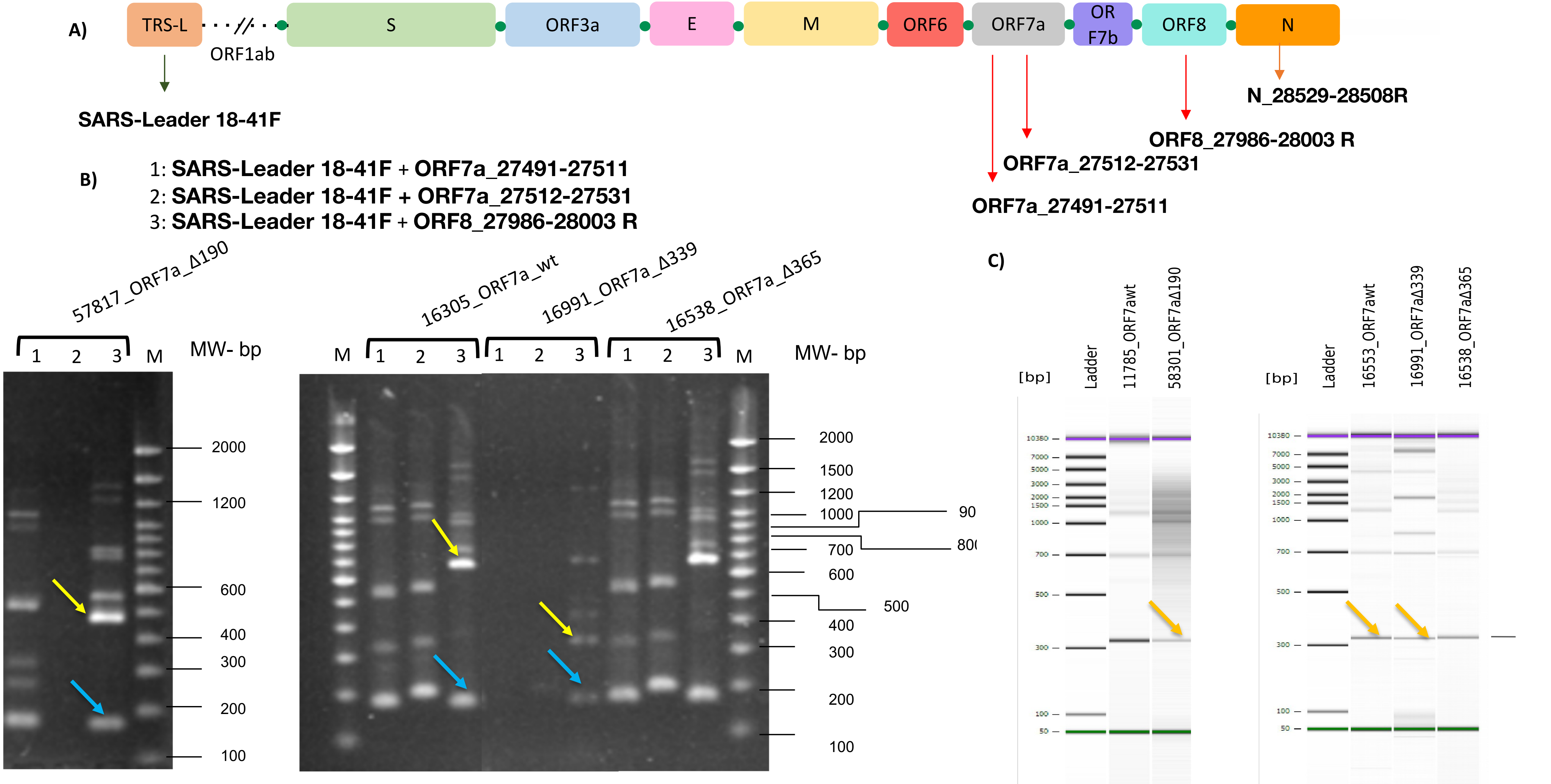

Figure S3

A phylogenetic analysis of SARS-CoV-2 samples complete genome sequences, high coverage, available on GISAID, from april to september of 2020, in the State of Rio de Janeiro.

Groups: familial core (blue - bootstrapping 98% of confidence); coworkers (red - bootstrapping of 92%) and samples from Uruguay (green).

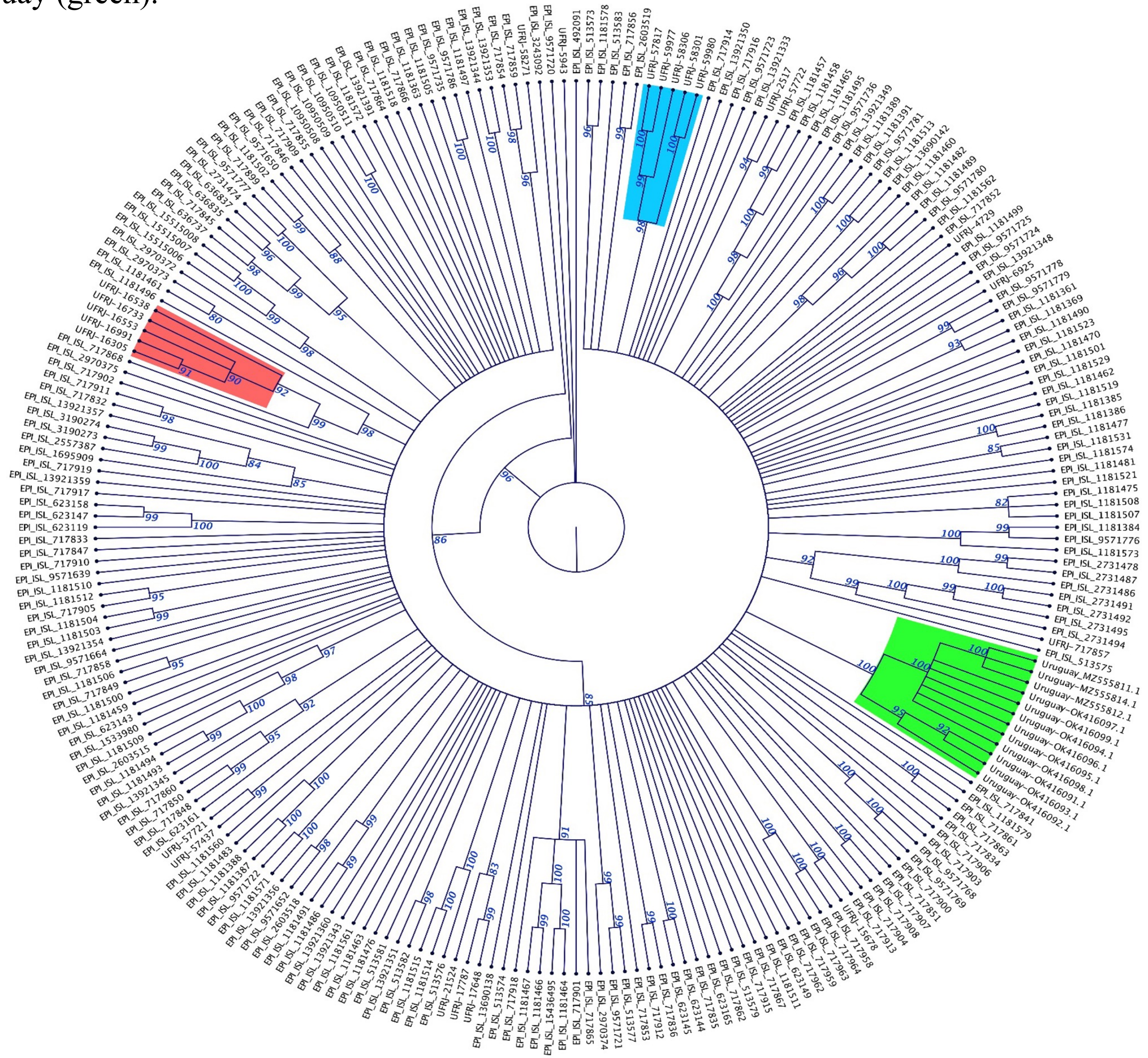

Table S1 Clinical information of family members with ORF7a_Δ190 nt

Sample ID	GISAID accession ID/NCBI accession ID	Date of birth	Gender	Date of sampling	Ct	Age (years)	Relationship	Information gathered on sampling date
57817_ORF7aΔ190	EPI_ISL_16093 686/ OQ430703).	01/22/1976	male	05/27/2020	21,22	44	father	Mild symptoms: head and bodyache and fever; no hospitalization needed
58301_ORF7aΔ190	EPI_ISL_16093 682/ OQ430699	4/5/2011	female	06/01/2020	13,8	9	Daughter	Mild symptoms: head and bodyache and fever; no hospitalization needed
58306_ORF7aΔ190	EPI_ISL_16093 685/ OQ430702	08/19/1977	female	06/01/2020	20,18	43	mother	Mild symptoms: headache and runny nose, no hospitalization needed
59977_ORF7aΔ190	EPI_ISL_16093 683/ OQ430700	09/30/2011	female	06/09/2020	18,98	8	niece	Mild symptoms: cough and runny nose; no hospitalization needed
59980_ORF7aΔ190	EPI_ISL_16093 684/ OQ430701	02/20/2013	male	06/09/2020	24,54	7	son	Mild symptoms: cough and runny nose; no hospitalization needed

Table S2 Nucleotide mutations and amino acid substitutions in genomes of SARS-CoV-2 with ORF7a_Δ190 nt

**Sample**	Nucleotide Substitutions											Amino Acid Substitutions								
**59977 Δ 190**	C4014T		C4543T	C14408T	C21855T	A23403G	T27299C	G28881A	G28882A	G28883C	T29148C	ORF1a:T1250I		ORF1b:P314L	ORF6:I33T	S:S98F	S:D614G	N:R203K	N:G204R	N:I292T
**59980 Δ 190**	C4014T		C4543T	C14408T	C21855T	A23403G	T27299C	G28881A	G28882A	G28883C	T29148C	ORF1a:T1250I		ORF1b:P314L	ORF6:I33T	S:S98F	S:D614G	N:R203K	N:G204R	N:I292T
**58306 Δ 190**	C4014T		C4543T	C14408T	C21855T	A23403G	T27299C	G28881A	G28882A	G28883C	T29148C	ORF1a:T1250I		ORF1b:P314L	ORF6:I33T	S:S98F	S:D614G	N:R203K	N:G204R	N:I292T
**57817 Δ 190**	C4014T	T4118C	C4543T	C14408T	C21855T	A23403G	T27299C	G28881A	G28882A	G28883C	T29148C	ORF1a:T1250I	ORF1a:Y1285H	ORF1b:P314L	ORF6:I33T	S:S98F	S:D614G	N:R203K	N:G204R	N:I292T
**58301 Δ 190**	C4014T		C4543T	C14408T	C21855T	A23403G	T27299C	G28881A	G28882A	G28883C	T29148C	ORF1a:T1250I		ORF1b:P314L	ORF6:I33T	S:S98F	S:D614G	N:R203K	N:G204R	N:I292T
	**ORF 1ab**				**S**		**ORF6**	**N**				**ORF1ab**			**ORF6**	**S**		**N**		

Table S3 Clinical information of coworkers with ORF7a_Δ339 and ORF7a_Δ365 deletion and ORF7a_wt

Sample ID	GISAID accession ID/NCBI accession ID	Gender	Date of sampling	Ct		Relationship	Information gathered on sampling date
				N2	RP		
16538_ORF7aΔ 365	EPI_ISL_159775 91/ OQ430693	male	7/29/2020		25.36	Coworkers	Mild symptoms, no hospitalization needed
16553_ORF7a_ wt	EPI_ISL_160936 80	male	7/29/2020		20.57	Coworkers	Mild symptoms, no hospitalization needed
16991_ORF7aΔ 339	EPI_ISL_159775 92/ OQ430694)	male	8/4/2020	29.09		Coworkers	Mild symptoms, no hospitalization needed
16305_ORF7a_ wt	EPI_ISL_159775 90/ OQ430692	unknown	7/27/2020	17.48		Coworkers	Mild symptoms, no hospitalization needed

Table S4 Nucleotide mutations and amino acid substitutions in genomes of SARS-CoV-2 with ORF7a_Δ339 and ORF7a_Δ365 deletion and ORF7a_wt

Sample	Nucleotide Substitutions															Amino Acid substitutions								
16553_wt	C241T	C3037T	C4099T	A12082T	C14408T	G22014A	T22015G	G22017T	A23403G	A24292G	T27299C	G28881A	G28882A	G28883C	T29148C	K1202Q	P314L	S151K	W152L	D614G	I33T	R203K	G204R	I292T
16305_wt	C241T	C3037T	C4099T	A12082T	C14408T				A23403G	A24292G	T27299C	G28881A	G28882A	G28883C	T29148C		P314L			D614G	I33T	R203K	G204R	I292T
16538 Δ 365 & wt	C241T	C3037T	C4099T	A12082T	C14408T				A23403G	A24292G	T27299C	G28881A	G28882A	G28883C	T29148C		P314L			D614G	I33T	R203K	G204R	I292T
16991 Δ 399	C241T	C3037T	C4099T	A12082T	C14408T				A23403G	A24292G	T27299C	G28881A	G28882A	G28883C	T29148C		P314L			D614G	I33T	R203K	G204R	I292T
	**ORF1ab**					**S**					**ORF6**	**N**				**ORF1ab**		**S**			**ORF6**	**N**		

Table S5

Plaque purify 16538 ORF7a_Δ365 and 16538 ORF7a_wt stock viruses grown in different cell cultures.

Sample	Virus type	qPCR (C_T)	Cell type	Passage
P2	16538 ORF7a_wt	22.00	**Vero E6**	2
P4	16538 ORF7a_wt	22.35		
P5	16538 ORF7a_Δ365	23.19		
P6	16538 ORF7a_Δ365	21.96		
P2	16538 ORF7a_wt	28.58	**Vero ACE2 TMPRSS2**	3
P4	16538 ORF7a_wt	28.27		
P5	16538 ORF7a_Δ365	30.38		
P6	16538 ORF7a_Δ365	28.28		
P2	16538 ORF7a_wt	und	**Calu-3**	3
P4	16538 ORF7a_wt	25.25		
P5	16538 ORF7a_Δ365	24.65		
P6	16538 ORF7a_Δ365	24.37		
